# A Positively Selected *MAGEE2* LoF Allele Is Associated with Sexual Dimorphism in Human Brain Size and Shows Similar Phenotypes in *Magee2* Null Mice

**DOI:** 10.1093/molbev/msab243

**Published:** 2021-08-31

**Authors:** Michał Szpak, Stephan C Collins, Yan Li, Xiao Liu, Qasim Ayub, Marie-Christine Fischer, Valerie E Vancollie, Christopher J Lelliott, Yali Xue, Binnaz Yalcin, Huanming Yang, Chris Tyler-Smith

**Affiliations:** 1 Wellcome Sanger Institute, Wellcome Genome Campus, Hinxton, United Kingdom; 2 Inserm UMR1231, Genetics of Developmental Disorders Laboratory, University of Bourgogne Franche-Comté, Dijon, France; 3 IGBMC, UMR7104, Illkirch, Inserm, France; 4 BGI-Shenzhen, Shenzhen, China; 5 Tsinghua Shenzhen International Graduate School, Tsinghua University, Shenzhen, China; 6 Monash University Malaysia Genomics Facility, School of Science, Bandar Sunway, Selangor Darul Ehsan, Malaysia

**Keywords:** loss of function, positive selection, brain size, mouse knockout, sexual dimorphism, MRI

## Abstract

A nonsense allele at rs1343879 in human *MAGEE2* on chromosome X has previously been reported as a strong candidate for positive selection in East Asia. This premature stop codon causing ∼80% protein truncation is characterized by a striking geographical pattern of high population differentiation: common in Asia and the Americas (up to 84% in the 1000 Genomes Project East Asians) but rare elsewhere. Here, we generated a *Magee2* mouse knockout mimicking the human loss-of-function mutation to study its functional consequences. The *Magee2* null mice did not exhibit gross abnormalities apart from enlarged brain structures (13% increased total brain area, *P *=* *0.0022) in hemizygous males. The area of the granular retrosplenial cortex responsible for memory, navigation, and spatial information processing was the most severely affected, exhibiting an enlargement of 34% (*P *=* *3.4×10^−6^). The brain size in homozygous females showed the opposite trend of reduced brain size, although this did not reach statistical significance. With these insights, we performed human association analyses between brain size measurements and rs1343879 genotypes in 141 Chinese volunteers with brain MRI scans, replicating the sexual dimorphism seen in the knockout mouse model. The derived stop gain allele was significantly associated with a larger volume of gray matter in males (*P *=* *0.00094), and smaller volumes of gray (*P *=* *0.00021) and white (*P *=* *0.0015) matter in females. It is unclear whether or not the observed neuroanatomical phenotypes affect behavior or cognition, but it might have been the driving force underlying the positive selection in humans.

## Introduction

Gene inactivation is often considered selectively disadvantageous, and has been studied mainly in the clinical context of lethality and disease. Loss of a nonessential gene function might, however, have no impact on an organism’s fitness (selective neutrality), or in some rare instances might even provide opportunities for adaptation (MacArthur and Tyler-Smith 2010; Narasimhan et al. 2016). There are numerous documented examples of gene pseudogenization fixed in the human lineage, which equipped our ancestors with advantageous phenotypes unique to our species (Wang et al. 2006). These include reduction of masticatory muscles due to *MYH16* inactivation by a frameshifting mutation (Stedman et al. 2004) or a human-specific exon deletion/frameshift mutation in the human *CMAH* linked to malaria resistance in vitro (Martin et al. 2005) and successfully studied in vivo using mouse models of human evolution (Hedlund et al. 2007; Okerblom et al. 2018). In the human-like *Cmah* inactivation, delayed wound healing and age-related hearing loss were initially detected (Hedlund et al. 2007), and subsequently increased running endurance in mice, suggesting that pseudogenization of this gene might have turned our ancestors into marathon runners (Okerblom et al. 2018). This case also illustrates the potential pleiotropic effects of loss-of-function alleles and the importance of comprehensive functional studies using model organisms where the environment and genetic background are controlled (Enard 2014).

Apart from the fixed human-specific loss-of-function mutations underlying the interspecies differences between humans and other primates, there are few examples of loss-of-function alleles segregating in the human population, and subjected to local selective pressures contributing to human genetic differentiation. Classical examples of this kind include alleles conferring immune resistance to pathogens, such as near-complete pseudogenization of *CASP12* outside Africa due to increased resistance to severe sepsis (Xue et al. 2006), the stop-gained variant in *FUT2* (rs601338, also known as *se^428^*) linked to rota- and norovirus resistance, found at high frequencies in Africans (49%) and Europeans (44%) but absent in East Asians (Kelly et al. 1995; Ferrer-Admetlla et al. 2009) and finally, a frameshift deletion in *CCR5* (rs333 or Δ32) found at 11% in Europe and manifesting AIDS resistance, but hypothesized to have undergone positive selection due to previous infectious diseases, albeit disputed by others (Sabeti et al. 2005).

It is worth noting that the loss of function can result from disruption of either the coding sequence or the gene regulation, like the Duffy O blood group null allele conferring *vivax* malaria resistance and almost fixed in most African populations (1000 Genomes Project Consortium et al. 2015). A regulatory variant in the 5′-UTR of the *ACKR1* locus (rs2814778) abolishes promotor activity and expression of the Duffy blood group antigen by disrupting the binding site for the GATA1 transcription factor (Tournamille et al. 1995; Iwamoto et al. 1996). Nonfixed adaptive loss-of-function variation has also been studied functionally using mouse models. A stop-gain allele (rs1815739) in *ACTN3* encoding the fast skeletal muscle fiber protein α-actinin-3, found at high frequency outside Africa, has been shown to be overrepresented in endurance runner athletes (Yang et al. 2003; Lee et al. 2016) and linked to improved cold tolerance (Wyckelsma et al. 2021). This association has been experimentally validated in *Actn3* mouse knockouts, which were shown to run 33% further than wild-type mice likely due to more efficient aerobic muscle metabolism (MacArthur et al. 2007, 2008).

The handful of adaptive variants linked to experimentally validated selected phenotypes contrast sharply with thousands of putatively positively selected alleles of unclear function (Szpak et al. 2018, 2019). One such example is a known positively selected premature stop codon causing ∼80% protein truncation (rs1343879; ENST00000373359.4:c.358G>T; ENSP00000362457.2:p.Glu120Ter) of the poorly studied *MAGEE2* gene on human chromosome X (Yngvadottir et al. 2009; Szpak et al. 2018). It is one of the strongest examples of positive selection in East Asia, with the selected allele found at 84% frequency across the region and in the Americas, but only at low frequency elsewhere, yet with no understanding of its function and the reasons for selection (Yngvadottir et al. 2009; Szpak et al. 2018). Here, we investigate the functional consequences of the naturally occurring human *MAGEE2* knockout and suggest reasons for its selection. We first generated *Magee2* null mice mimicking the human loss-of-function allele and performed comprehensive in vivo and postmortem phenotyping, and then replicated our findings in a follow-up association study in human cohorts.

## Results

### Primary Whole-Body Mouse Phenotyping

A mouse knock-out was generated by CRISPR/Cas9-mediated critical exon deletion (supplementary fig. S1, Supplementary Material online) in the C57BL/6N background. The resulting *Magee2^em1(IMPC)Wtsi^* mice were then phenotyped. At weaning age, mouse survival was assessed from successfully genotyped mice originating from several different litters, showing the expected number of mice. The standardized primary phenotyping, encompassing a wide set of phenotypic tests both in vivo and after terminal necropsy (White et al. 2013) (supplementary appendix, Supplementary Material online) did not reveal any abnormalities: the *Magee2* null mice were phenotypically grossly normal. Male and female mice were weighed the same day each week from 4 until 16 weeks of age and did not show body weight phenotype (supplementary fig. S2, Supplementary Material online). Subsequently, guided by the brain-specific *MAGEE2* expression in humans (GTEx Consortium 2015), we performed a detailed neuroanatomical phenotyping of mutant mouse brains.

### Secondary Neuroanatomical Phenotyping of Mutant Mouse Brains

**Fig. 1.**
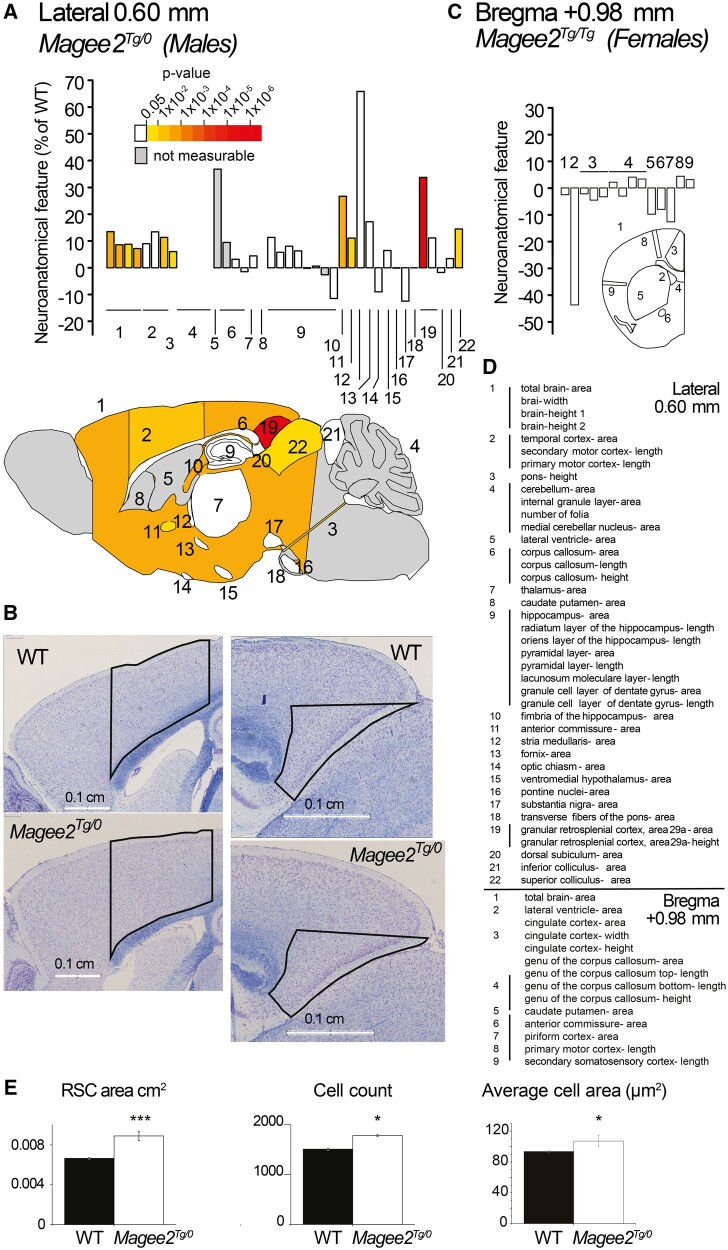
*Magee2* male hemizygous mice exhibit enlarged brain structures. (*A*) Histograms show neuroanatomical features as the percentage decrease (minus scale) or increase (plus scale) of the measured brain regions in *Magee2* mutant male mice compared with matched controls (0%). The bottom image shows a schematic representation of affected brain regions in mutant male mice at 16 weeks of age plotted in the sagittal plane (section at Lateral +0.60 mm) and colored according to *P* values above. Gray indicates parameters which could not be confidently tested (*n* too low to calculate the statistics). The name of each brain region numbered in (*A*) is given in panel (*D*). (*B*) Representative sagittal brain sections of *Magee2* hemizygous male mice and matched controls double-stained with Nissl and Luxol fast blue, showing the motor and retrosplenial granular cortices. (*C*) On a coronal plane at Bregma +0.98 mm, female homozygous mutant mice show a trend toward reduced structure sizes compared with controls, albeit not significant. Histograms and plane representation as in (*A*). (*D*) Details of brain regions assessed in order of appearance in panels (*A*) and (*C*) together with corresponding numbers. The description of the parameters used is also provided in supplementary file 1 and figure S4, Supplementary Material online. (*E*) Cellular phenotyping: left, the granular retrosplenial cortex total area; middle, total cell count; right, average cell area.

We examined the brain anatomy of adult *Magee2* hemizygous male and homozygous null female mice using parasagittal and coronal histo-phenotyping, respectively (Mikhaleva et al. 2016; Collins et al. 2018). Slides scanned to cell level resolution (supplementary fig. S3, Supplementary Material online) were used to quantify 40 brain morphological parameters across 22 distinct brain structures in males from a parasagittal brain section at the plane Lateral +0.60 mm, and 14 parameters across nine unique brain structures in females from a coronal section at Bregma +0.98 mm (fig. 1*D*, supplementary fig. S4, Supplementary Material online, raw data provided in supplementary file 1, Supplementary Material online). This quantification was blind to the genotype.

Mild to severe brain anomalies reminiscent of macrocephaly were detected in male hemizygous mice and are summarized in figure 1*A*. Ten parameters were significantly enlarged in *Magee2* mutant male mice when compared with matched wild-type controls, including the total brain area (+13%, *P *=* *0.0022), the width of the brain (+9%, *P *=* *0.0019), the height of the brain (rostral: +9%, *P *=* *0.023; caudal: +7%, *P *=* *0.0031), the fimbria of the hippocampus (+27%, *P *=* *0.0024), the area of the anterior part of the anterior commissure (+11%, *P *=* *0.035), the height of the pons (+6%, *P *=* *0.028), the area of the superior colliculus (+14%, *P *=* *0.011) and the height of the primary motor cortex (+11%, *P *=* *0.0055). Interestingly, the area of the granular retrosplenial cortex was the most severely affected brain structure, exhibiting an enlargement of +34% (*P *=* *3.4×10^−6^) (fig. 1*B*). This region is responsible for memory, navigation, and spatial information processing (Pothuizen et al. 2010; Powell et al. 2017). Taken together, these results suggest that *Magee2* is involved in the regulation of brain size, notably of the cortices and the commissures. In female homozygous knockout mice (fig. 1*C*), however, no difference was observed compared with controls although the general trend was toward reduction of structure sizes, especially for the lateral ventricle (−43%, not significant). Our results suggest sexual dimorphism in the neuroanatomy of *Magee2* null mice.

Cellular phenotyping in males (fig. 1*E*) revealed that although the granular retrosplenial cortex total area is significantly enlarged +34% (*P *=* *3.6×10^−6^), this can be attributed to a combined increase in the cell count (+18%, *P *=* *0.01) and larger cell size as measured by the Nissl coloration (cell body area estimated from the nucleus and Nissl bodies) by +14% (*P *=* *0.045) (raw data provided in supplementary file 1, Supplementary Material online). As a result, the measured cell density in mutant males was lower than in WT controls (−12%, *P *=* *0.004).

### Association between *MAGEE2* Inactivation and Brain Measures in Humans

Encouraged by the neuroanatomical phenotypes in null mice and the expression patterns of the functional *MAGEE2* allele in human brain (GTEx Consortium 2015), we performed an association study between brain volume quantified from MRI scans and rs1343879 genotypes in a group of 141 Han Chinese volunteers (Females *N* = 74, Males *N* = 67, supplementary tables S1–S3, Supplementary Material online) from Shenzhen, China. We confirm that the derived A allele at rs1343879 causing ∼80% MAGEE2 truncation is found at high frequencies across Asia (South–North gradient) and the Americas, and shows a strong signature of positive selection (supplementary fig. S5, Supplementary Material online). The brain measurements included the absolute and relative cerebral gray matter, white matter, and cerebrospinal fluid volumes, as well as the average cortical thickness. We also performed quantification of 68 distinct brain regions (supplementary file 2, Supplementary Material online). Linear regression was performed to assess the association between the rs1343879 genotype and brain measures, adjusting for age, sex, and body height.

**Fig. 2.**
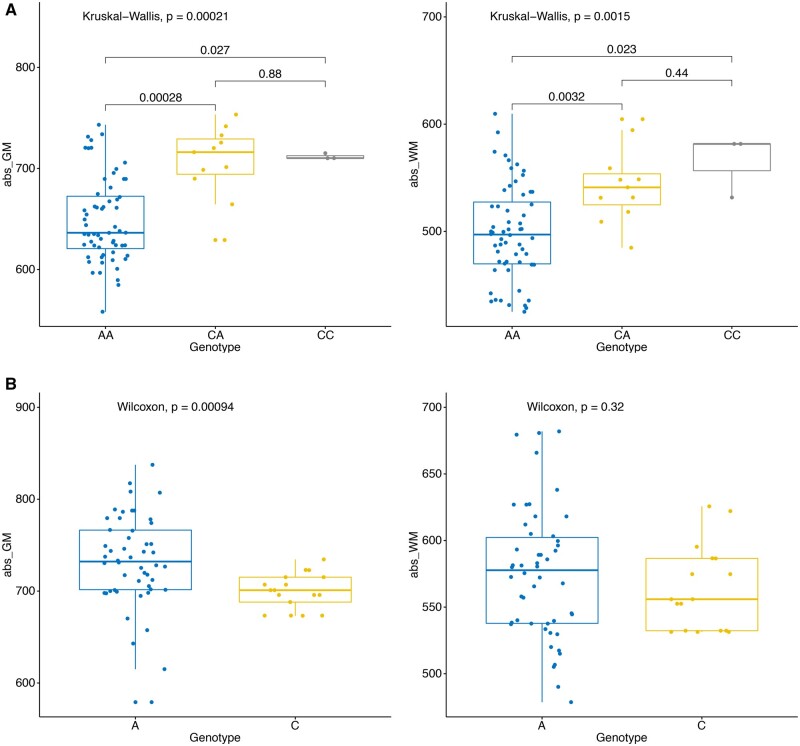
Association between rs1343879 genotypes and cerebral gray and white matters in human. A is the derived allele causing premature stop gain, whereas C is the ancestral allele. (*A*) Significant differences of gray (left) and white (right) matter among different genotype groups in females. (*B*) Comparison of gray (left) and white (right) matter among different genotype groups in males.

All the brain size measures were significantly different between males and females. Interestingly, females carrying the homozygous derived AA genotype had smaller gray and white matters than those carrying CA and CC genotypes (fig. 2*A*). In contrast, in hemizygous males, the effect is opposite: individuals carrying the derived A allele have relatively larger gray matters (*P* = 0.0009), no significant difference of white matter between the two genotypes were found (fig. 2*B*). We thus replicated the sexual dimorphism in the direction of the *Magee2* inactivation effect on brain size seen in the mouse model.

Considering the above sex differences, we tested the genetic association in a linear model by adding age, sex, and body height as well as genotype and sex interaction as covariates (formula = abs_WM/abs_GM ∼ Geno + Age + Sex + body height + Geno×Sex). The results show that rs1343879 was significantly associated with absolute volumes of gray (*P* = 0.000138) and white matter (*P* = 0.00254), and that the sex and genotypes have a significant interaction (gray matter: *P* = 0.00610; white matter: *P* = 0.02563).

The human brain regions most affected by the rs1343879 genotype (largest effect sizes measured by the regression coefficient) were the inferior, middle and superior frontal gyrus, precentral gyrus, superior parietal gyrus, orbito-frontal gyri, lateral occipital lobe, inferior lateral parietal lobe, and posterior temporal lobe (supplementary file 2, Supplementary Material online). The effect sizes observed in males and females were either opposite or not significantly different from zero. As the segmentation of human and mouse brains was different, we could not directly test the difference in the granular retrosplenial cortex volume between rs1343879 genotypes in humans. However, a larger section of the human brain containing posterior regions adjacent to the hippocampus (including the granular retrosplenial cortex) defined as the posterior temporal lobe, was one of the human brain regions, whose volume was significantly associated with rs1343879 genotype (lPosTeLo *P *=* *0.017 in the combined sample, the genotype by sex interaction was near-significant FDR *P *=* *0.067; supplementary file 2, Supplementary Material online). These measurements were not significant in the smaller sample stratified by sex, although the left posterior temporal lobe exhibited a trend toward increased volume in males carrying the derived A allele (near significant *P *=* *0.066), and the opposite direction was observed in females.

## Discussion

This is, to our knowledge, the first report linking the inactivation of *MAGEE2* in humans and its mouse ortholog to the enlargement of brain structures. Such a finding resulting from a loss of gene function with no detectable accompanying phenotypes in mouse knockouts is rare (Collins et al. 2019). Collins et al. (2019) analyzed over 1,500 mouse mutants, identifying around 200 genes whose disruptions yielded neuroanatomical phenotypes. Only seven of those resulted in significant enlargement of the total brain area, namely *Cep41* (+19%), *Sytl1* (+19%), *Pik3cb* (+16%), *Sparc* (+16%), *Ifi27* (+15%), *Herc1* (+13%), and *Efcab3*-like (+7%). Mouse knockouts characterized by megalencephaly often have strong accompanying phenotypes (e.g., *Herc1*, *Cep41*, and *Efcab3*-like). Interestingly, similarly to *MAGEE2* inactivation selected in East Asia, two of these genes also bear signatures of positive selection in humans. Recent studies have suggested that human *HERC1* has been subject to local positive selection in East Asia, as indicated by marked differences in allele and haplotype frequencies between East Asians and non-East Asians, together with low genetic diversity in East Asia (Yuasa et al. 2009; Szpak et al. 2018). Similarly, disruption of *Efcab3*-like (Gm11639, ENSMUSG00000040838) in mouse resulted in brain size enlargement, suggesting a potential role of *Efcab3*-like in regulation of brain size and development (Lilue et al. 2018). This largely conserved gene is disrupted in gorilla (*Gorilla gorilla*) and human (*Homo sapiens*) by a recombination event (∼15 Mb intrachromosomal rearrangement), which split it into two separate protein-coding genes, *EFCAB3* and *EFCAB13*, suggesting a possible old selection event in the Hominine lineage (Lilue et al. 2018). Based on the available ancient DNA data, the derived A allele for rs1343879 in human *MAGEE2* appears to be old, as it is observed in a ∼45,000-year-old Ust’-Ishim man from Siberia (Fu et al. 2014) and is also presently found in Africa, the Middle East, and Europe, albeit at low frequency (supplementary fig. S5, Supplementary Material online). The selection in East Asians must have happened after the split of basal Eurasians, and most likely acted on a segregating allele, rather than a de novo mutation.

What was the basis for selection on *MAGEE2* inactivation in East Asia? The lack of detectable pleiotropic phenotypes in the null mouse, together with gene expression restricted almost exclusively to brain tissues in humans (GTEx Consortium 2015) and no indication of alternative splicing of this single exon gene, suggests that the observed neuroanatomical phenotypes might have been the driver of selection. As brain morphogenesis is a complex process contributing to higher order cognition (Collins et al. 2019), further studies are needed to investigate *MAGEE2* allele effects on cognition. It is important to stress that the observed increase of brain size in males does not necessarily imply cognitive consequences. It might even be that the brain enlargement impacts skull morphometrics, and that this inactivation has been selected due to reasons unrelated to cognition, such as sexual selection. It is, therefore, currently difficult to suggest specific consequences of this inactivation which drove it to such high frequency in East Asia. There are, however, studies linking *Magee2* to neuronal plasticity (forming new neuronal connections) in rat (Nartey et al. 2020). Pinpointing possible behavioral or cognitive implications of this neuroanatomical phenotypes is limited due to scarce evidence, nonetheless the brain area particularly affected by *Magee2* inactivation in mouse is the granular retrosplenial cortex, responsible for object recency memory, navigation, and spatial information processing (Pothuizen et al. 2010; Powell et al. 2017).

Another outstanding question relates to the different phenotypic manifestations of *MAGEE2* inactivation in males and females in both human and mouse, and their implications for the reasons for positive selection in humans. It is difficult to suggest why selection would favor opposite effects in males and females without further cognitive evidence, but it might be that the selective advantage only applies to one sex. Furthermore, even though we observed a similar trend of decreased brain size in females in both human and mouse, the effect sizes were different, with a pronounced effect in human and no statistical significance in mouse. Although humans and mice share neurodevelopmental principles, it could be that this discrepancy arises from differences in the brain organization between the two species, such as lack of the complex cortical folding in mouse, contrasting with the human brain (Collins et al. 2019). Although sexual dimorphism in mammalian brain-related traits is well-established (Karp et al. 2017), the molecular mechanism by which *MAGEE2* regulates brain morphogenesis differently in males and females requires further investigation. The fact that this gene is located on a sex chromosome might, however, be relevant here. It is worth adding that sex chromosomes have been generally excluded from previous genome-wide association studies identifying common genetic variants explaining intracranial volume, so the importance of variation in *MAGEE2* has been overlooked to date (Hibar et al. 2015; Adams et al. 2016).

Even though future studies are needed to address some of the questions raised here, this is the first study addressing the functional consequences of one of the strongest signals of a classic hard sweep in East Asia, linking it to neuroanatomical phenotypes characterized by sexual dimorphism in brain morphogenesis in human and mouse. As the number of classical hard sweeps linked to causal variants with known phenotypes is tiny (Szpak et al. 2019), this study provides an additional, functionally validated, example of local adaptation associated with phenotypic changes driving human interpopulation diversification.

## Materials and Methods

### Mutant Mouse Generation

*Magee2* was targeted using CRISPR/Cas9-mediated critical exon deletion (Shen et al. 2013; Boroviak et al. 2016) (supplementary table S4, Supplementary Material online) as part of the Sanger Institute Mouse Genetics Project, part of the International Knockout Mouse Consortium (IKMC) resource (allele name: *Magee2^em1(IMPC)Wtsi^*). The deletion was designed to span 1,566 bp, making up 86% of the *Magee2* coding sequence (a single exon gene supported by cDNA evidence; supplementary fig. S1, Supplementary Material online). Four guide RNAs (two at either 5′ and 3′ end of the CE region, supplementary table S4, Supplementary Material online) were designed using the WTSI Genome Editing (WGE) tool (Hodgkins et al. 2015) and microinjected together with Cas9 mRNA (Trilink) into the cytoplasm of single-cell C57BL/6N zygotes. The injected embryos were transferred to oviduct of postcoital pseudopregnant C57BL/6N female recipients. The progeny were screened as described below to confirm the engineered allelic structure.

#### Genotyping by End-Point PCR

Mice were genotyped using a combination of separate PCR reactions that detect the gene-specific wild-type allele and a mutant allele-specific short range PCR, followed by agarose gel electrophoresis (supplementary table S5 and fig. S1, Supplementary Material online). Primers and reagents used in the PCR reaction are listed in supplementary tables S6 and S7, Supplementary Material online, and amplification conditions can be found in supplementary table S8, Supplementary Material online.

#### Genotyping by Loss of WT Allele qPCR Assay (Gene-Specific Assay)

The wild-type loss of allele (LoA) qPCR assay with a hydrolysis probe assay (Applied Biosystems TaqMan technology) was used to determine the copy number of the wild-type allele in a sample. The primers used (Life Technologies) are described in supplementary table S9 and figure S1, Supplementary Material online. The number of copies of the wild-type allele was detected using a FAM-labeled custom qPCR TaqMan assay. These were multiplexed with a VIC-labeled endogenous control assay (TaqMan Copy Number Reference Assay, Mouse, Tfrc; Applied Biosystems Part No. 4458366). Reference DNA controls of known genotypes were included to facilitate correct analysis. Reactions are performed in a 10 μl volume (supplementary table S10, Supplementary Material online) using an Applied Biosystems 7900HT Fast Real-Time PCR System or Applied Biosystems Viia7 with DNA prepared using the Sample-to-SNPTM kit (Applied Biosystems) from mouse ear biopsies and GTXpressTM buffer (Applied Biosystems). The amplification conditions are given in supplementary table S11, Supplementary Material online.

### Animal Husbandry and Primary Phenotyping

Mice were housed in a specific-pathogen-free facility with sentinel monitoring at standard temperature (19–23 °C) and humidity (55 ± 10%), on a 12 h dark, 12 h light cycle (07:30–19:30, no twilight period) and fed a standard rodent chow diet (Mouse Breeder Diet 5021, Labdiet). Food and water were available ad libitum for most of the pipeline. The mice were housed for phenotyping in groups of three to four mice per cage with Aspen bedding substrate, standard environmental enrichment of a nestlet, and a cardboard tunnel. The standardized primary phenotyping, encompassing a set of phenotypic tests covering 215 clinical parameters, was applied to cohorts of seven mutant males, seven mutant females, and matched controls (seven males and seven females per week). This high-throughput screen can be divided into three general categories: developmental, in vivo (reproduction, infection and immunity, musculoskeletal system, metabolism, and endocrinology), and necropsy with blood analysis, described in detail elsewhere (White et al. 2013). All animals were regularly monitored for health and welfare and were additionally checked before and after procedures. A list of all measured parameters can be found in supplementary appendix, Supplementary Material online. The care and use of mice in the study were carried out in accordance with UK Home Office regulations, UK Animals (Scientific Procedures) Act of 1986 under a UK Home Office license (P77453634) that approved this work, which was reviewed regularly by the WTSI Animal Welfare and Ethical Review Body.

### Secondary Neuroanatomical Studies in Mouse

All steps of the neuroanatomical studies were performed with experimenters blinded to the animals’ genotypes. Standard operating procedures are described in more details elsewhere (Mikhaleva et al. 2016; Collins et al. 2018). Mouse brain samples were immersion-fixed in 10% neutral buffered formalin for 48 h, before paraffin embedding and sectioning at 5 μm thickness using a sliding microtome (Leica RM 2145). Sagittal section was stereostatically defined as the plane Lateral +0.60 mm, coronal section was collected at Bregma +0.98 mm according to the Allen Mouse Brain Atlas (Sunkin et al. 2013). Different planes were used for males and females due to biobanked material availability, but these were shown to be comparable (Mikhaleva et al. 2016; Collins et al. 2018, 2019). Brain sections were double-stained using luxol fast blue for myelin and cresyl violet for neurons, and scanned at cell-level resolution using the Nanozoomer whole-slide scanner 2.0HT C9600 series (Hamamatsu Photonics, Shizuoka, Japan) (supplementary fig. S3, Supplementary Material online). Covariates, for example, sample processing dates and usernames were collected at every step of the procedure using in-house ImageJ plugins and used to identify data drifts. This image analysis pipeline was also used to capture and standardize measurements of brain areas and lengths. Each image was quality controlled for the accuracy of sectioning relative to the reference atlas and controlled for asymmetries and histological artifacts.

Forty brain morphological parameters (including 25 area and 14 length measurements, and the number of cerebellar folia) were measured on the parasagittal section in males (supplementary file 1 and fig. S4, Supplementary Material online), resulting in the quantification of the following 22 unique brain structures at Lateral +0.60 mm (fig. 1*A*): 1) the total brain area; 2) the primary and secondary motor cortices; 3) the pons; 4) the cerebellar area, the internal granular layer of the cerebellum and the medial cerebellar nucleus; 5) the lateral ventricle; 6) the corpus callosum; 7) the thalamus; 8) the caudate putamen; 9) the hippocampus and its associated features; 10) the fimbria of the hippocampus; 11) the anterior commissure; 12) the stria medullaris; 13) the fornix; 14) the optic chiasm; 15) the hypothalamus; 16) the pontine nuclei; 17) the substantia nigra; 18) the fibers of the pons; 19) the granular retrosplenial cortex; 20) the dorsal subiculum; 21) the inferior colliculus; and 22) the superior colliculus.

In females, a coronal section was used at Bregma +0.98 mm and resulted in the quantification of nine unique brain structures comprising 14 brain morphological parameters. Brain structures assessed were 1) the total brain area; 2) the lateral ventricles; 3) the Cingulate cortex; 4) the genu of the corpus callosum; 5) the caudate putamen; 6) the anterior commissure; 7) the piriform cortex; 8) the primary motor cortex; 9) the secondary somatosensory cortex. The measures overlays are shown in supplementary figure S4, Supplementary Material online. All samples were also systematically assessed for cellular ectopia (misplaced neurons).

Depending on the type of sections studied, parasagittal or coronal sections, statistical analyses were carried out using either a linear mixed model (LMM) developed in R using PhenStat (Kurbatova et al. 2015), a package providing a variety of statistical methods for the analysis of large-scale phenotypic associations from the International Mouse Phenotyping Consortium (IMPC), or student two-tailed equal variance test (*t*-tests). Controls were either local (wild-type animals from the same production line, matched for age [16 weeks], sex and background [B6N]), and/or littermate controls. In males, 114 local age/sex/background-matched control mice and three hemizygous *Magee2* knockouts (16 weeks old) were analyzed; in females, four local 16-week-old background-matched controls (including one littermate control) were obtained and compared with three homozygous knockout mice (raw data available in supplementary file 1, Supplementary Material online). When parasagittal sections were not available and the number of mouse lines not sufficient to use a LMM, a *t*-test was used. Cell counts were measured from slides using ImageJ Macro with automatic cell segmentation and compared using a *t*-test.

### Association Study in Humans

141 Han Chinese volunteers (female *N* = 74, male *N* = 67) from the local area in Shenzhen, China were recruited. A written informed consent form was signed by each individual. Whole-genome sequencing (WGS) to 30× coverage was conducted from DNA in white cells using the BGI-seq500.

WGS data were aligned and variants called by the Picard (http://github.com/broadinstitute/picard/releases/tag/2.19.2, last accessed August 20, 2019)/BWA (Li and Durbin 2009)/GATK (DePristo et al. 2011) pipeline. SNPs with mapping quality greater than 40, sequencing depth greater than 4, variant quality greater than 2.0, Phred score of Fisher’s test *P* value for stand bias smaller than 60.0, Haplotype score smaller than 13.0 and distance of alternative allele from the end of reads greater than 8.0 were kept for the following analyses. One individual among relatives within 3rd degree of relationship was randomly selected to keep in the clean data set. SNP rs1343879 genotypes were extracted from the clean data set and used in the current study.

Structural MRI scans were acquired at a field strength of 3.0 Tesla with a T1‐weighted magnetization‐prepared rapid gradient‐echo (MPRAGE) sequence (voxel size = 1 × 1 × 1.25 mm^3^; FoV = 257×257 mm^2^; slide number = 192; TR = 2,530.0 ms; TE = 3.0 ms) utilizing a Siemens Prisma scanner.

All images were processed with the CAT12 toolbox (http://www.neuro.uni-jena.de/cat/, last accessed August 20, 2019, version r1109) within SPM12 (http://www.fil.ion.ucl.ac.uk/spm/software/spm12/, last accessed August 20, 2019, version 6225) using MATLAB (8.3) to gather brain size measurements. The absolute and relative cerebral gray matter, white matter, and cerebrospinal fluid volumes were quantified. The average cortical thickness was calculated.

Linear regression was performed to assess the association between genotype and brain measures. Age, sex, and body height were adjusted as covariants in the model. To assess sex differences in brain size and their relationship with genetic factors, the samples were stratified into male and female groups. In addition, the sex and genotype unit was added, to test whether they interact. Wilcoxson tests were used to demonstrate the significance levels.

The automatic segmentation of brain MRIs into 68 regions of interest (ROI) was performed according to the Hammers’ Brain Atlas (Hammers et al. 2003; Gousias et al. 2008). The regression model used in ROI analysis was ROI_volume ∼ Geno + Age + Sex + Body Height + Geno×Sex for the combined sample, and ROI_volume ∼ Geno + Age + Body Height for the samples stratified by sex.

## Supplementary Material

Supplementary data are available at *Molecular Biology and Evolution* online.

## Supplementary Material

msab243_Supplementary_Data
