# Supplementary material for: A Positively Selected MAGEE2 LoF Allele Is Associated with Sexual Dimorphism in Human Brain Size and Shows Similar Phenotypes in Magee2 Null Mice
Source: Mol Biol Evol. 2021 Aug 31;38(12):5655–63. doi: 10.1093/molbev/msab243 (PMC8662591; doi:10.1093/molbev/msab243)
Supplement: msab243_Supplementary_Data [file msab243_supplementary_data.zip › Supplementary Material.pdf]

Supplementary Material

Appendix

List of parameters measured in the primary phenotyping of the *Magee2* null mice with no significant departure from normality:

Homozygous viability at P14

Homozygous Fertility

Weight Curves:
- Weight Curve
- Body Weight AUC (wk4-14)

Neurological Assessment:
- Tremor
- Transfer Arousal
- Gait (inc. Ataxia)
- Tail Elevation
- Startle Response
- Positional Passivity
- Trunk Curl
- Limb Grasping
- Pinna touch reflex
- Corneal touch reflex
- Contact Righting Reflex
- Headbobbing/Circling
- Convulsions

Dysmorphology:
- Head Coat/Hair Presence
- Head Coat/Hair Colour
- Head Coat/Hair Colour Pattern
- Head Coat/Hair Appearance
- Head Coat/Hair Length
- Dorsal Coat/Hair Presence
- Dorsal Coat/Hair Colour
- Dorsal Coat/Hair Colour Pattern
- Dorsal Coat/Hair Appearance
- Dorsal Coat/Hair Length
- Ventral Coat/Hair Presence
- Ventral Coat/Hair Colour
- Ventral Coat/Hair Colour Pattern
- Ventral Coat/Hair Appearance
- Ventral Coat/Hair Length
- Limb Coat/Hair Presence
- Limb Coat/Hair Colour

- Limb Coat/Hair Colour Pattern
- Limb Coat/Hair Appearance
- Limb Coat/Hair Length
- Paw Coat/Hair Presence
- Paw Coat/Hair Colour
- Paw Coat/Hair Colour Pattern
- Paw Coat/Hair Appearance
- Paw Coat/Hair Length
- Digit Coat/Hair Presence
- Digit Coat/Hair Colour
- Digit Coat/Hair Colour Pattern
- Digit Coat/Hair Appearance
- Digit Coat/Hair Length
- Tail Coat/Hair Presence
- Tail Coat/Hair Colour
- Tail Coat/Hair Colour Pattern
- Tail Coat/Hair Appearance
- Tail Coat/Hair Length
- Pinna Skin Appearance
- Pinna Skin Colour
- Dorsal Skin Appearance
- Dorsal Skin Colour
- Ventral Skin Appearance
- Ventral Skin Colour
- Limb Skin Appearance
- Limb Skin Colour
- Paw Footpad Skin Appearance
- Paw Footpad Skin Colour
- Tail Skin Appearance
- Tail Skin Colour
- Fight Wound(s)
- Head Morphology
- Head Size
- Eyelid Opening
- Eye Morphology
- Snout Morphology
- Snout Size
- Vibrissae
- Vibrissae Size
- Vibrissae Colour
- Vibrissae Shape
- Mouth Morphology
- Incisors
- Incisors Morphology
- Incisor Colour
- Pinna
- Pinna Size

- Pinna Morphology
- Pinna Position
- Pinna Rotation
- External Genitalia
- External Genitalia Morphology
- External Genitalia Size
- Forelimb
- Forelimb Size
- Forelimb Morphology
- Forepaw
- Forepaw Size
- Forepaw Morphology
- Forepaw Footpad Size
- Forepaw Digit Count (normal =10)
- Forepaw Digit Size
- Forepaw Digit Morphology
- Forepaw Digit Fusion
- Forepaw Nail Count
- Forepaw Nail Length
- Forepaw Nail Morphology
- Forepaw Nail Colour
- Hindlimb
- Hindlimb Size
- Hindlimb Morphology
- Hindpaw
- Hindpaw Size
- Hindpaw Morphology
- Hindpaw Footpad Size
- Hindpaw Digit Count (normal =10)
- Hindpaw Digit Size
- Hindpaw Digit Morphology
- Hindpaw Digit Fusion
- Hindpaw Nail Count
- Hindpaw Nail Length
- Hindpaw Nail Morphology
- Hindpaw Nail Colour
- Tail
- Tail Length
- Tail Morphology
- Head Skin Appearance
- Head Skin Colour

Glucose Tolerance (IP Glucose Tolerance Test Short Fast):

- ip-GTT
- Fasted Plasma Glucose

Body Composition (DEXA, Ultrafocus 100):

- Bone Mineral Density

- Bone Mineral Content
- Bone Area
- Body Weight
- Nose to Tailbase Length
- Estimated Total Tissue Mass
- Lean Mass
- Fat Mass
- Fat Percentage Estimate
- Total sample area
- Soft tissue mass

Plasma Chemistry (Ketamine / Xylazine):
- Sodium
- Potassium
- Chloride
- Glucose
- Fructosamine
- Triglycerides
- Cholesterol
- High Density Lipoprotein
- Low Density Lipoprotein
- NEFAC
- Glycerol
- Total Thyroxine (THYRX)
- Amylase
- Alanine Aminotransferase
- Alkaline Phosphatase
- Creatine Kinase
- Aspartate Aminotransferase
- Total Bilirubin
- Total Protein
- Albumin
- Creatinine
- Urea
- Calcium
- Magnesium
- Iron
- Phosphate
- Insulin (MSD)

Haematology Terminal (Vet abc Plus +):
- White Blood Cell Count
- Red Blood Cell Count
- Mean Corpuscular Volume
- Haemoglobin
- Haematocrit
- Mean Corpuscular Haemoglobin
- Mean Corpuscular Haemoglobin Conc.

- Red Blood Cell Distribution Width
- Platelet Count
- Mean Platelet Volume

Peripheral Blood Leukocytes Terminal (PBL flow cytometry assay 16 week LSR II 2 panel whole blood):

- Total T cell percentage
- Total T cell number
- ab T cell %
- alpha beta T cell number
- CD4+ ab T cell %
- CD4+ ab T cell number
- CD4+ ab Treg cell %
- CD4+ ab Treg cell number
- CD4+ ab Teff cell %
- CD4+ ab Teff cell number
- CD4+ KLRG1+ ab T cell %
- CD4+ KLRG1+ ab T cell number
- CD8+ ab T cell %
- CD8+ ab T cell number
- CD8+ ab Teff cell %
- CD8+ ab Teff cell number
- CD8+ KLRG1+ ab T cell %
- CD8+ KLRG1+ ab T cell number
- gd T cell %
- gd T cell number
- NKT cell percentage
- NKT cell number
- NK cell percentage
- NK cell number
- KLRG1+ NK cell %
- KLRG1+ NK cell number
- B cell percentage
- B cell number
- IgD+ B cell %
- IgD+ B cell number
- Neutrophil percentage
- Neutrophil number
- Eosinophil percentage
- Eosinophil number
- Monocyte percentage
- Monocyte number
- Ly6Chigh monocyte percentage
- Ly6Chigh monocyte number
- Ly6Clow monocyte percentage
- Ly6Clow monocyte number

Micronuclei T109:

- % MN-NCE
- % MN-RET
- % RET

Supplementary figures

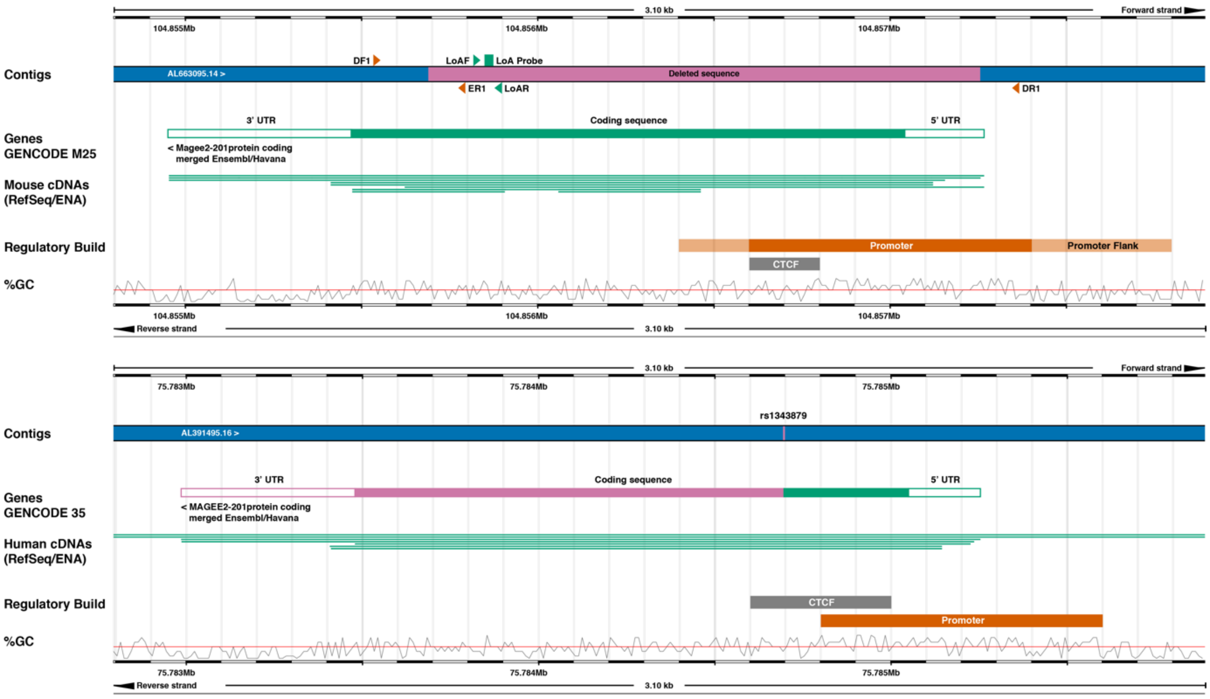

Figure S1. **Comparison of the human and mouse MAGEE2/Magee2 loci**. Human *MAGEE2* and its mouse orthologue share 85% cDNA sequence identity. cDNA expression evidence for both genes is shown as green lines. **Top**: Ensembl *Mus musculus* version 101.38 (GRCm38.p6) Chromosome X: 104,854,798 – 104,857,894. Critical exon deletion spanning 86% of Magee2 CDS is indicated in pink. End-point PCR and LoA qPCR primers are show as arrows. **Bottom**: Ensembl *Homo sapiens* version 101.38 (GRCh38.p13) Chromosome X:75,782,795 –75,785,891. The naturally occurring truncation of 77% of the human *MAGEE2* CDS is shown in pink. Modified after Ensembl [1].

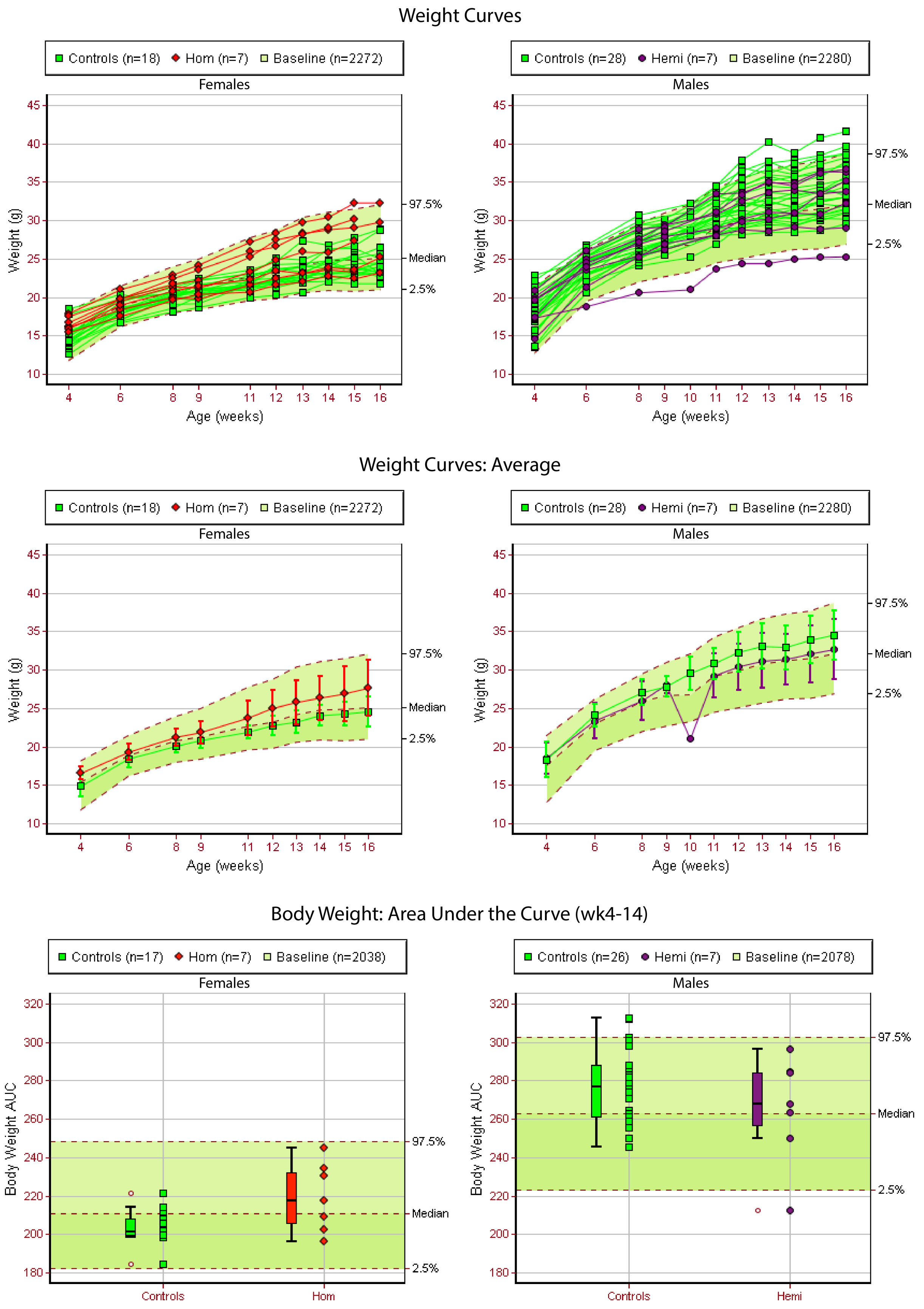

Figure S2. Null Magee2 mouse mutant weight curves. Mice were fed on Mouse Breeder Diet (5021, Labdiet) from weaning and weighed between 4 and 16 weeks of age.

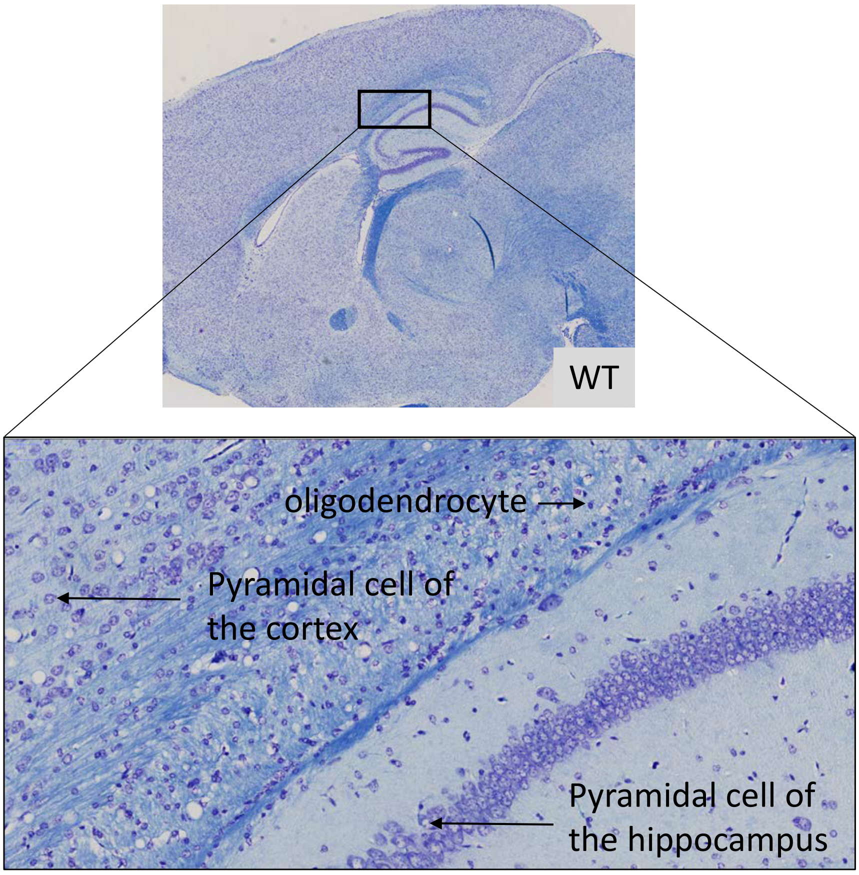

Figure S3. Representative scan of mouse brain section.
Example of a mouse brain section double-stained with the luxol fast blue for myelin and the cresyl violet for neurons scanned at cell-resolution using the Nanozoomer whole-slide scanner 2.0HT C9600 series.

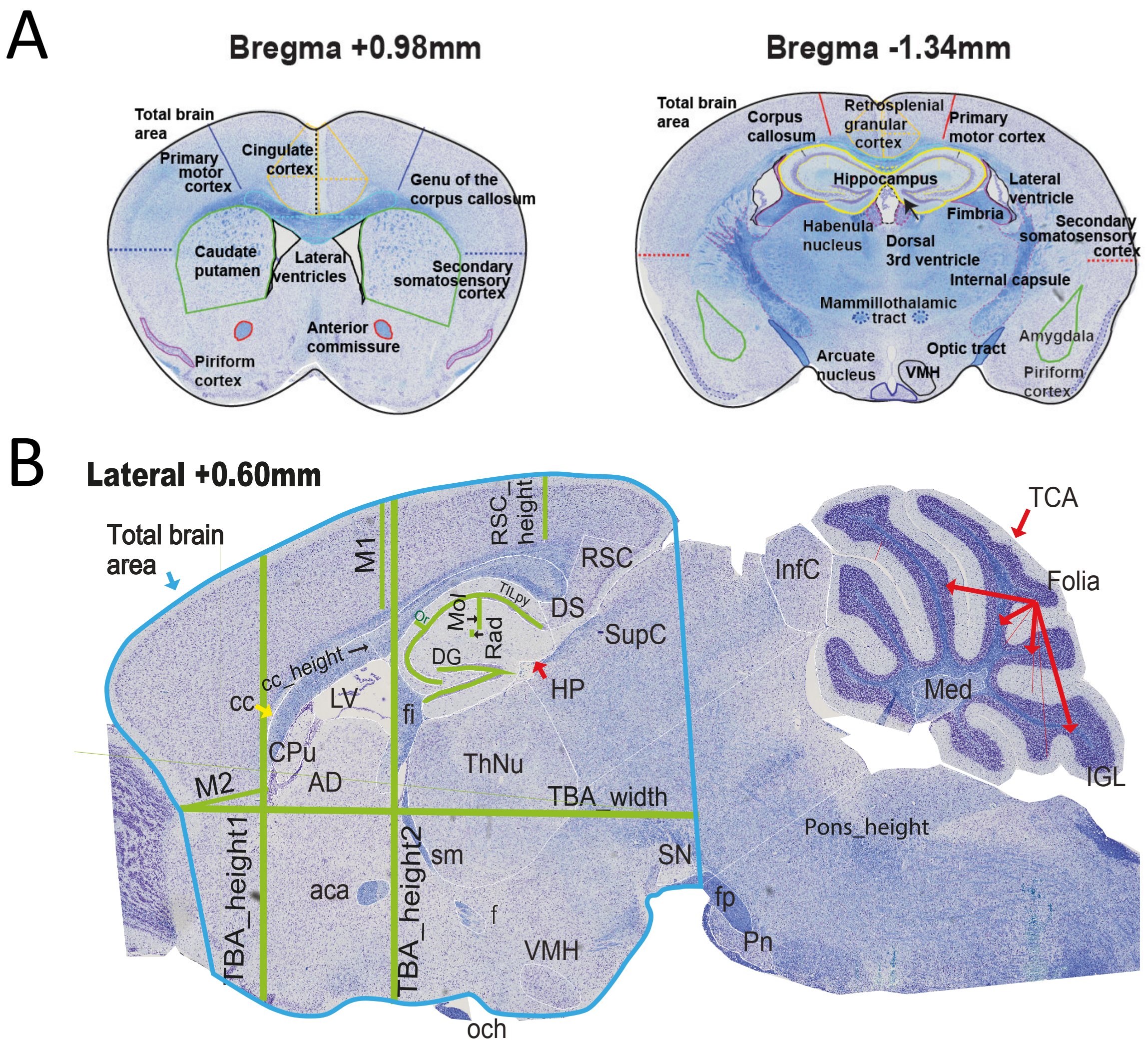

Figure S4. Overlay of brain morphological parameters measured.
Brain morphological parameters across distinct brain structures measured in females on a coronal section at Bregma +0.98 mm (**A left**) and in males from a parasagittal brain section at the plane Lateral +0.60 mm (**B**). Coronal Bregma -1.34 mm section shown for comparison (**A right**).

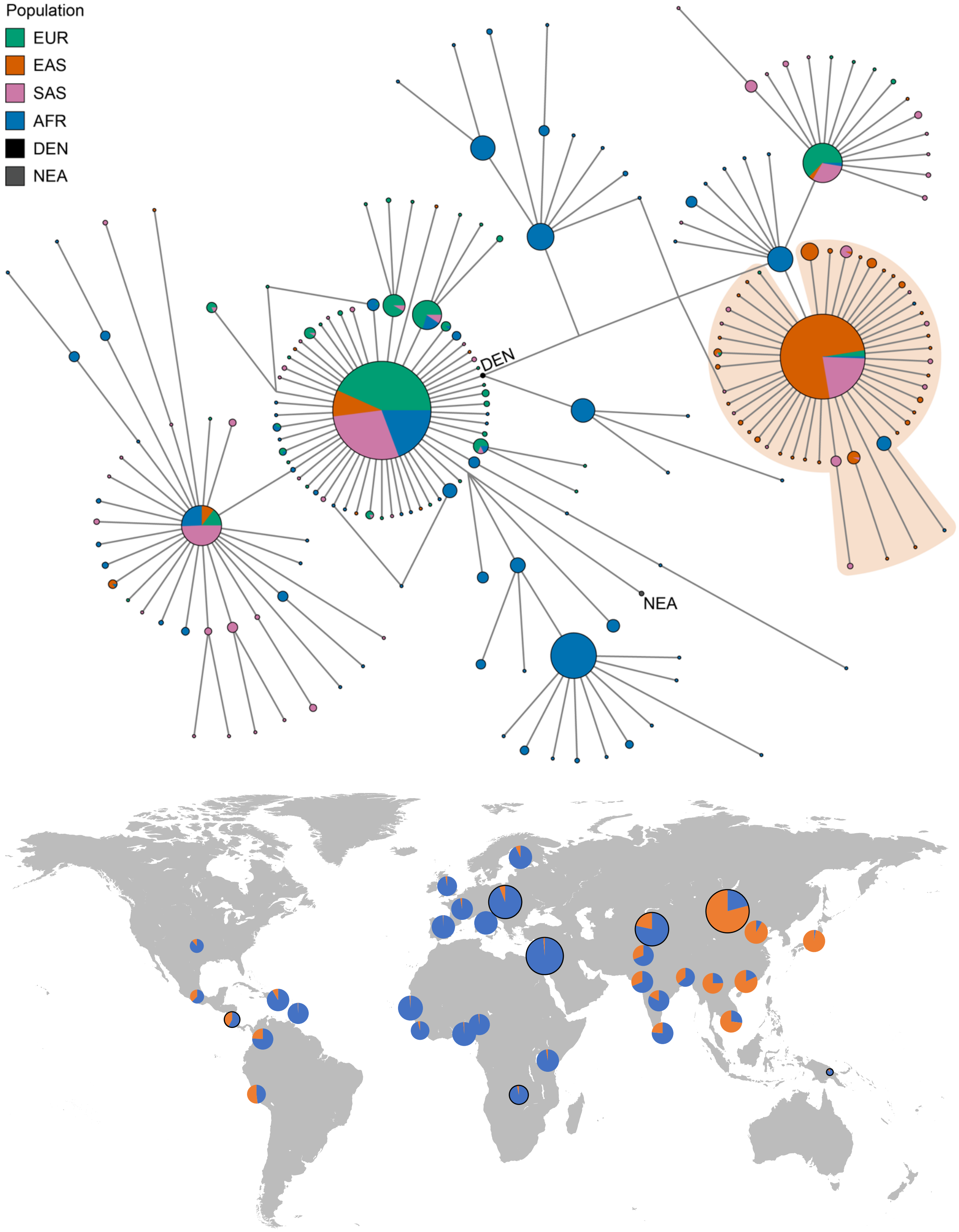

Figure S5. Worldwide allele frequency at rs1343879 and haplotype structure of the surrounding genomic region.

Top: Haplotype structure in *MAGEE2.* The median-joining haplotype network was generated using Network 5.0.1.1 for all SNPs within a 10 kb region surrounding rs1343879. Each circle represents a distinct haplotype. Circle area is proportional to haplotype frequency; the branch length shows number of mutational steps between haplotypes (shortest line equals one step). The network was generated for 2157

individuals from the 1000 Genomes Project, Phase 3 [2] of African (AFR), East (EAS) and South Asian (SAS), and European (EUR) ancestry individuals, plus the high-coverage Denisova (DEN) [3] and Neanderthal (NEA) genomes [4]. Haplotypes are coloured by population frequency. rs1343879 derived haplotypes are highlighted in orange.

Bottom: Allele frequency at rs1343879 in worldwide human populations. rs1343879 allele frequency in The 1000 Genomes Project Phase 3 subpopulations [2] and HGDP continental groups (black outline: Africa, America, Central and South Asia, East Asia, Europe, Middle East, Oceania) [5]. The ancestral C allele is shown in blue; the derived A allele in orange. Pie charts are proportional to the sample size (number of X chromosomes). In The 1000 Genomes Project data, the derived A allele is found at ~28% in South Asia and ~85% frequency in East Asia following a north-south gradient, with the selected allele reaching its highest frequency of 97% in Japan.

Supplementary tables

Table S1. Association study summary statistics in the whole sample (males and females combined).

	Mean	SD
Age	33.25	5.17
Grey matter (cm^3)	690.50	56.37
White matter (cm^3)	538.40	55.68

Table S2. Association study summary statistics in females.

	Mean	SD	
Age	32.51	4.67	
Grey matter (cm^3)	659.90	46.40	
White matter (cm^3)	508.0	46.35	
**rs1343879 genotype**	**AA**	**CA**	**CC**
count	60	11	3

Top: Mean age, cerebral grey and white matter volumes and the corresponding standard deviations (SD). Bottom: rs1343879 genotype counts.

Table S3. Association study summary statistics in males.

	Mean	SD
Age	34.06	5.60
Grey matter (cm^3)	724.4	46.25
White matter (cm^3)	572.00	44.92
**rs1343879 genotype**	**A**	**C**
count	50	17

Top: Mean age, cerebral grey and white matter volumes and the corresponding standard deviations (SD). Bottom: rs1343879 genotype counts.

Table S4. Guide RNAs used in initial CRISPR/Cas9 experiments

Sequence	Chr	Start position	End position
CCTACGCAGGCGCACTCAAGCTC	X	104857251	104857273
CCTAGCTTTGCCGCATCCTACGC	X	104857235	104857257
GCGTTGCCTCATTAGCTTACAGG	X	104855700	104855722
CCTGCATTCTAGGTAGCGTTGCC	X	104855685	104855707

Table S5. Standard PCRs primer pairs and expected size bands for end-point PCR genotyping

Assay	Forward Primer	Reverse Primer	Expected Size Band (bp)
Wild-type	Magee2_DF1	Magee2_ER1	261
Mutant	Magee2_DF1	Magee2_DR1	267

Table S6. End-point PCR genotyping primer sequences

Primer Name	Primer Sequence (5' > 3')
Magee2_DF1	CACAGCCTCCCTGTATTGCT
Magee2_ER1	CGTGTCAAAGAGGCCAAGAT
Magee2_DR1	ATAAGTTCGAACCCCACAGC

Table S7. End-point PCR genotyping reaction setup

Reagent	µl
DNA (~50-100 ng)	1
10x Buffer	1.5
$MgCl_2$ (50 mM)	0.45
Platinum Taq (Invitrogen)	0.15
dNTPs (100 mM)	0.15
Primer 1 (10 µM)	0.3
Primer 2 (10 µM)	0.3
ddH20	11.15
Total	15

Table S8. End-point PCR genotyping amplification conditions

Step	Conditions	Time
1	94°C	5 min
2	94°C	30 sec
3	58°C	30 sec
4	72°C	1:30 sec
5	Go to '2' + 34 cycles	-
6	72°C	5 min
7	12°C	forever

Table S9. Primers for LoA qPCR assay

Primer Type	Primer Sequence (5' > 3')
Forward Primer	CCCAGGATTGGCATCAAGTA
Reverse Primer	CCCAACCAGAATCAGAAGAAGA
Probe Primer	TCTGCCCAGGCTCTCTAGCATTACT

Table S10. LoA qPCR reaction setup

Reagent	µl
2x GTXpressTM buffer	5
20x target assay	0.5
ddH2O	3
Tfrc endogenous 20x assay	0.5
DNA	1

Table S11. LoA qPCR Amplification conditions

Step	Conditions	Time
1	95°C	20 sec
2	95°C	10 sec
3	60°C	30 sec
4	Go to '2' + 34 cycles	-

Supplementary References

1. Yates, A.D., Achuthan, P., Akanni, W., Allen, J., Allen, J., Alvarez-Jarreta, J., Amode, M.R., Armean, I.M., Azov, A.G., Bennett, R., et al. (2020). Ensembl 2020. Nucleic acids research 48, D682-D688.
2. Genomes Project, C., Auton, A., Brooks, L.D., Durbin, R.M., Garrison, E.P., Kang, H.M., Korbel, J.O., Marchini, J.L., McCarthy, S., McVean, G.A., et al. (2015). A global reference for human genetic variation. Nature 526, 68-74.
3. Meyer, M., Kircher, M., Gansauge, M.T., Li, H., Racimo, F., Mallick, S., Schraiber, J.G., Jay, F., Prufer, K., de Filippo, C., et al. (2012). A high-coverage genome sequence from an archaic Denisovan individual. Science 338, 222-226.
4. Prufer, K., Racimo, F., Patterson, N., Jay, F., Sankararaman, S., Sawyer, S., Heinze, A., Renaud, G., Sudmant, P.H., de Filippo, C., et al. (2014). The complete genome sequence of a Neanderthal from the Altai Mountains. Nature 505, 43-49.
5. Bergstrom, A., McCarthy, S.A., Hui, R., Almarri, M.A., Ayub, Q., Danecek, P., Chen, Y., Felkel, S., Hallast, P., Kamm, J., et al. (2020). Insights into human genetic variation and population history from 929 diverse genomes. Science 367.
